# Applying MLP-Mixer and gMLP to Human Activity Recognition

**DOI:** 10.3390/s25020311

**Published:** 2025-01-07

**Authors:** Takeru Miyoshi, Makoto Koshino, Hidetaka Nambo

**Affiliations:** 1 Graduate School of National Science and Technology, Kanazawa University, Kanazawa 920-1192, Japan; 2 National Institute of Technology, Ishikawa College, Tsubata 929-0392, Japan

**Keywords:** human activity recognition, multi-layer perceptrons (MLPs), MLP-mixer, gMLP, smartphone, inertial measurement unit (IMU)

## Abstract

The development of deep learning has led to the proposal of various models for human activity recognition (HAR). Convolutional neural networks (CNNs), initially proposed for computer vision tasks, are examples of models applied to sensor data. Recently, high-performing models based on Transformers and multi-layer perceptrons (MLPs) have also been proposed. When applying these methods to sensor data, we often initialize hyperparameters with values optimized for image processing tasks as a starting point. We suggest that comparable accuracy could be achieved with fewer parameters for sensor data, which typically have lower dimensionality than image data. Reducing the number of parameters would decrease memory requirements and computational complexity by reducing the model size. We evaluated the performance of two MLP-based models, MLP-Mixer and gMLP, by reducing the values of hyperparameters in their MLP layers from those proposed in the respective original papers. The results of this study suggest that the performance of MLP-based models is positively correlated with the number of parameters. Furthermore, these MLP-based models demonstrate improved computational efficiency for specific HAR tasks compared to representative CNNs.

## 1. Introduction

Human activity recognition (HAR) using inertial measurement unit (IMU) data has gained widespread adoption in healthcare [1,2] and fitness [3] tracking because of its lower implementation complexity than vision-based systems and reduced privacy concerns for users. IMUs integrated into modern smartphones and smartwatches have been utilized for advanced safety features, including detecting vehicular collisions and falls and initiating emergency calls when necessary. The enhancement of sensor data recognition accuracy is crucial for these safety applications’ widespread adoption and effectiveness in real-world scenarios. As this field of research advances and enables accurate collection of various daily activity data, it becomes possible to calculate daily calorie expenditure and physical activity levels. Applications that provide personalized daily diet and exercise recommendations based on these data are expected to reduce health risks and extend healthy life expectancy. Advanced deep learning technology has facilitated the development of numerous HAR models, such as convolutional neural networks (CNNs) and recurrent neural networks (RNNs). HAR applications have shown promising results when adopting model architectures that excel in image recognition tasks. These adapted models demonstrate accuracy levels comparable to those in their original domain. However, in the field of image recognition, the landscape has evolved with the proposal of various non-convolutional models. These include the Vision Transformer [4], which relies entirely on Transformer architectures, and MLP-Mixer [5] and gMLP [6], both based exclusively on multi-layer perceptrons (MLPs). These models represent an alternative direction to conventional convolutional approaches in image recognition. We suggest that these non-convolutional models could be effectively applied to HAR tasks, potentially competing with traditional convolutional approaches. In the original MLP-based models, the number of parameters is tailored to image data based on the number of patches and channels. When applied to inertial measurement unit (IMU) sensor data, these models can achieve better accuracy with fewer parameters, as the number of patches and channels is typically smaller than in image data, assuming similar patch sizes. However, it should be noted that an excessive number of parameters might compromise the efficiency of the learning process. We applied two representative MLP-based models, MLP-Mixer and gMLP, to HAR tasks. Our evaluation focused on the performance of MLP-based models as we systematically reduced the number of parameters in their MLP layers and patch embedding dimensions (dim). The original number of parameters specified for the MLP-Mixer and gMLP models served as the baseline (100%) for a series of experiments in which we progressively reduced the number of parameters. These parameter reduction experiments showed that accuracy is proportional to the number of parameters. While this differs from our suggestion, we found that MLP-Mixer and gMLP demonstrate flexible scalability by adjusting the number of parameters. Our contributions through these experiments are as follows:

- We apply and evaluate MLP-based models (MLP-Mixer and gMLP) on HAR tasks, assessing their performance in terms of accuracy and computational efficiency, measured by the number of parameters. Additionally, we apply prominent CNN models originally proposed as image classification models to HAR and compare their results.
- We investigate the effects of parameter reduction by systematically examining the impact of decreasing the number of parameters in both MLP-Mixer and gMLP models. This analysis provides insights into the optimal level of parameter reduction when adapting MLP-based models for HAR applications. Furthermore, we clarified the FLOPs and actual training time for each model. These results serve as valuable metrics when evaluating models from an efficiency perspective. Our findings potentially lead to more efficient model designs in HAR.

## 2. Related Works

In sensor-based HAR, machine learning is used for activity classification [7,8,9], with reported cases using representative methods such as random forest (RF), support vector machine (SVM) and light gradient boosting machine (lightGBM). In recent years, as in other fields, deep learning has gained increased attention, and various deep learning models have been proposed [4,10,11,12,13]. In the field of sensor-based HAR, various deep learning models have been proposed [14,15,16,17]. Among the main approaches for constructing these models, we present two approaches and their current trends. One approach uses CNN-based models. CNNs are primarily employed for image classification; there have been reported cases of its successful application in HAR. A characteristic when applying these models to HAR is using one-dimensional kernels in the convolutional layers, as sensor data are one-dimensional. Approaches in model proposals include constructing shallow models specifically designed for HAR from scratch and adapting high-performing models from the image classification field to HAR applications. As reported by Zhong et al. [18], CNNs proposed for image classification have demonstrated good performance in the HAR domain as well. Based on these findings, this study takes an approach of applying new models proposed in the image classification field, specifically MLP-Mixer and gMLP, to the HAR domain and reports their performance. The second approach focuses on the one-dimensional time-series of sensor data, using RNN- and Transformer-based models that are also used in fields such as natural language processing. A recent trend in this approach is the proposal of HAR-specific models that combine with architectures such as CNN [19,20,21]. As in other fields, there is no definitive conclusion about which model is superior for HAR. Therefore, we consider it important to propose models through various approaches and evaluate and report their performance through experiments.

### 2.1. CNN-Based Models in HAR

CNN-based models, originally developed for image classification tasks, have been widely adopted and adapted for HAR applications. Early CNNs featured relatively shallow deep learning architectures with approximately one [14] to three [22,23] CNN layers. Yang et al. [23] accelerated processing by implementing bit operations instead of floating-point arithmetic, considering operation in mobile environments. To handle hierarchical class classification, Subasi et al. [24] proposed Branch CNNs (B-CNNs) consisting of six CNN layers. While models are typically constructed manually based on researchers’ expertise, Ismail et al. [15] adopted an approach that automatically searches for and constructs models suitable for HAR. Wang et al. [25] proposed models that combine CNN with attention mechanisms, which have been proven effective in other fields. Studies have also been conducted on adapting high-performing models from image classification to HAR [26]. Zhong et al. [18] evaluated the performance of various image classification models, ranging from VGG [10], Inception [27], and ResNet [11] to EfficientNet [12]. Their study revealed an interesting finding; EfficientNet, the state-of-the-art image classification model at the time, did not consistently outperform other models in HAR tasks. This outcome underscores the importance of empirical evaluation when applying models from other domains to HAR, regardless of their superiority in their original field.

### 2.2. RNNs and Attention-Based Models in HAR

Besides our approach of applying models proposed in the image classification field to HAR, approaches using RNN have also been explored, focusing on the one-dimensional time-series nature of sensor data. Murad et al. [16] introduced a model using Long Short-Term Memory (LSTM), while Li et al. [28] developed a Bidirectional LSTM approach. Recent advancements have led to hybrid models combining RNN-based components (such as Gated Recurrent Units (GRU) and LSTM) with CNNs. Ordóñez et al. [17] proposed the DeepConvLSTM model, which combines a four-layer CNN with two LSTM layers. Venkatachalam et al. [19] proposed a distinctive model with a three-layer structure where an LSTM layer is placed between two CNN layers in a CNN-LSTM-CNN arrangement. Models combining CNNs, attention, and GRU have been proposed [29], and Mim et al. [30] proposed a model that adopts the Inception module, originally introduced in Inception V2, as a CNN module. Thakur et al. [31] proposed ConvAE-LSTM, a model combining convolutional autoencoder with LSTM, and demonstrated that reconstruction through autoencoding improves accuracy in HAR. Besides end-to-end architectures, models using a two-stage approach that combine feature extraction and class classification have also been proposed. Tan et al. [32] proposed a model consisting of a feature extraction block that combines CNNs and BiGRU, and an output block that combines a single-hidden-layer feedforward neural network (SLFN) and regularized extreme machine learning (RELM) for recognition results. Praba et al. [20] proposed a model using CNN and LSTM for feature extraction, combined with SVM for class classification. Transformer-based models [13] have been adapted for HAR tasks [33,34,35]. These adaptations typically utilize the Transformer’s encoder component, similar to the Vision Transformer (ViT) [4] model proposed for image classification.

### 2.3. MLP-Based Models in HAR

In addition to CNN and RNN, studies have also explored the application of MLP to HAR. Kwapisz et al. [36] applied MLP to HAR before deep learning gained prominence. Their study reported favorable results when compared with other machine learning methods. A more complex architecture is the deep stacked multilayered perceptron (DS-MLP) [37]. This model combines five MLP-based base-learners and a meta-learner using stacked ensemble techniques. Nadia et al. [21] proposed a model that sequentially combines convolutional layers followed by MLP layers. Mao et al. [38] proposed GAM-MLP, which combines MLP and Transformer with their novel attention mechanism called the group attention module (GAM). These models were proposed specifically for HAR, rather than our approach of applying models originally proposed in the image recognition field.

## 3. Methods

### 3.1. Patch Embedding for Sensor Data Processing

**Figure 1:**
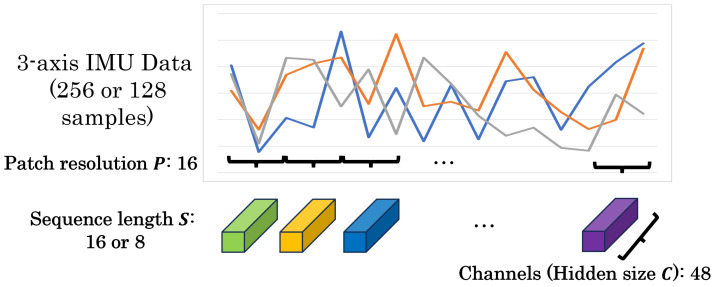
Applying of patch embedding to sensor data in HAR.

Patch embedding is a specific preprocessing step for MLP-Mixer and gMLP models, which involves dividing the input data into fixed-sized patches. In image processing, this typically means dividing an image into patch resolution (P, P)=(16, 16). However, the patching process differs for HAR applications using one-dimensional IMU sensor data, as in this study. Here, the time-series sensor data are divided into sequential patches, each with a resolution of P=16 time steps (Figure 1). In image processing, a (224, 224, 3) image divided into patches of resolution (16, 16) yields 196 patches (sequence length S=(224 ÷ 16) × (224 ÷ 16)). Each patch has a hidden size *C* of 768 channels (16 × 16 × 3), resulting in input dimensions of (196, 768) for the model. In the case of sensor data, with dimensions (256, 3) and a patch resolution of 16, the sequence length *S* is 16 (=256÷16), and the number of channels *C* per patch is 48 (=16×3). This results in input dimensions of (16, 48) for the model. The dimensions of the input data for sensor-based models vary depending on the original data size. For instance, when the sensor data dimension is (128, 3) with a patch resolution of 16, the resulting input has a sequence length of 8 and 48 channels per patch, leading to input dimensions of (8, 48). Given that the dimensionality of patched sensor data is significantly smaller than that of patched image data, we suggest that the number of MLP parameters in MLP-Mixer and gMLP models can be reduced for HAR tasks without significant performance loss.

### 3.2. MLP-Mixer

**Figure 2:**
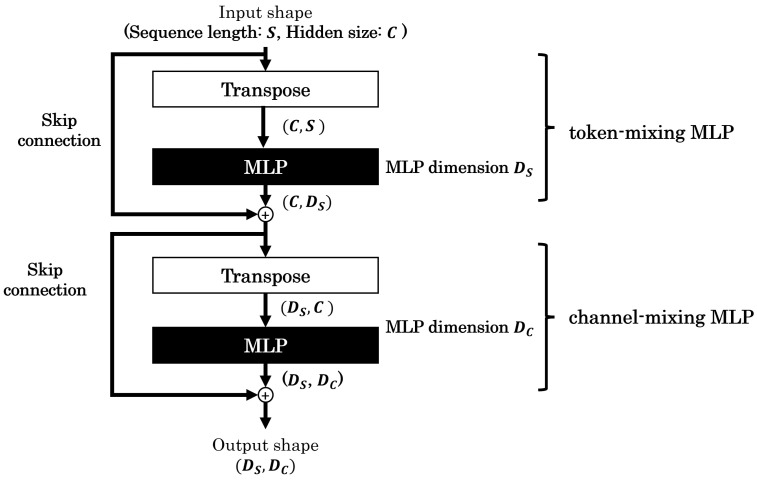
Architecture of MLP-Mixer for sensor data processing.

MLP-Mixer [5], proposed by Tolstikhin et al. in 2021, is an image classification model that eschews convolution and attention mechanisms, relying solely on MLPs. This model achieved comparable accuracy and inference performance to various state-of-the-art models of its time. The architecture of MLP-Mixer comprises Mixer layers, each combining two types of MLPs: token-mixing MLP and channel-mixing MLP. Figure 2 shows this structure, although, for simplicity, normalization and activation layers are omitted from the diagram. Token-mixing MLP extracts features by mixing information in the spatial direction between different tokens. Channel-mixing MLP extracts features by mixing information in the channel direction within the same token. The model is constructed by stacking multiple Mixer layers, each containing these two types of MLPs. The construction of the model requires hyperparameters:

- Patch resolution: *P*;
- Hidden size: *C*;
- The number of Mixer layers;
- The output dimension of the token-mixing MLP layer: $D_{S}$;
- The output dimension of the channel-mixing MLP layer: $D_{C}$.

In this study, we focus on reducing the parameters *C* (hidden size), $D_{S}$ (output dimension of token-mixing MLP), and $D_{C}$ (output dimension of channel-mixing MLP). For sensor data, the initial number of channels in each patch is 48. To achieve the desired hidden size, we adjust this using a convolution operation with a kernel size of 1. This convolution allows us to map the initial 48 channels to the specified number of channels required by the model’s architecture. The other parameters are maintained as specified in the original MLP-Mixer paper. This approach allows us to investigate the impact of dimensionality reduction on model performance in HAR tasks while preserving the fundamental structure of the model.

### 3.3. gMLP

**Figure 3:**
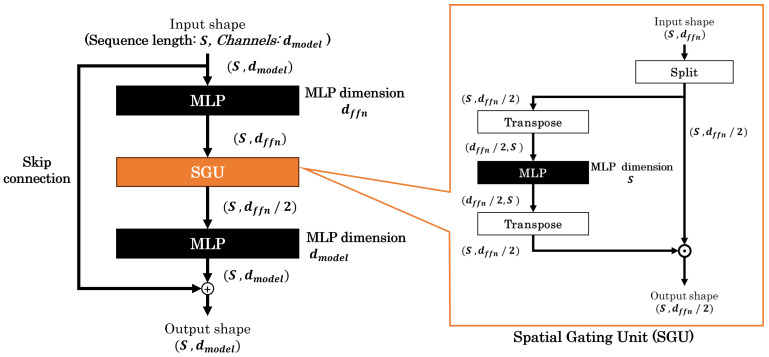
Architecture of gMLP for sensor data processing.

gMLP [6], proposed by Liu et al. in 2021, is an MLP-based deep learning model. It consists of gMLP blocks, which incorporate a structure called Spatial Gating Unit (SGU) alongside traditional MLPs. Figure 3 shows a gMLP block, with normalization and activation layers omitted for simplicity. The SGU, a vital component of this model, splits the input data into two parts along the channel direction and processes only one part through the MLP. This MLP extracts features from different tokens, similar to the token-mixing MLP in MLP-Mixer. The two parts are then multiplied before being passed to the next layer. A gMLP block contains two MLPs positioned around the SGU. These two MLPs perform a role similar to the channel-mixing MLP in MLP-Mixer, extracting features in the channel direction. The gMLP model is constructed by stacking multiple gMLP blocks. The construction of the model requires hyperparameters:

- Patch resolution: *P*;
- The number of gMLP blocks;
- The first MLP output dimensions in gMLP block: $d_{model}$;
  - $d_{model}$ is also used to calculate the number of channels for the input tokens (corresponding to *C* in MLP-Mixer).
- The second MLP output dimensions in gMLP block: $d_{ffn}$.

Similar to our approach with MLP-Mixer, we focus on reducing two specific parameters of gMLP: $d_{model}$ and $d_{ffn}$. The number of channels in each patch is converted to $d_{model}$ using a convolution with kernel size 1, similar to the approach used in MLP-Mixer. The other hyperparameters are maintained as specified in the original gMLP paper. This targeted parameter reduction allows us to investigate the impact on model performance in HAR tasks while preserving the fundamental structure of gMLP.

## 4. Experiments

### 4.1. Datasets

Our experiments utilize three distinct datasets with varying sampling frequencies: WISDM [36], UCI HAR [39], and HASC [40] (Table 1). Each dataset was collected using IMU sensors integrated into smartphones to record subject activities during specific predefined actions. These are all single-label, multi-class datasets. While the datasets share this common data collection approach, they differ in their sampling rates, number of activities recorded, and the specific smartphone placement on subjects. All three datasets focus on six common daily activities. We exclusively use the 3-axis accelerometer sensor data for consistency across datasets as our IMU input. HASC and WISDM are provided as continuous time series data for each activity from start to end. For these datasets, we set the window size to 256 along the time axis and extracted the data with a 50% overlap (stride = 128). This preprocessing resulted in a data dimension of (256, 3) for each extracted segment. On the other hand, UCI HAR is provided as pre-cut data with a window size of 128 and a stride of 64, resulting in a data shape of (128, 3). In this study, each extracted segment from all datasets was used as a single data point for the experiment. The window size of 256 was adopted as it is close to the common spatial resolution (224, 224) used in the CV field. This value has also been used in existing studies. For UCI HAR, the window size is 128 as the data dimensions are provided in a pre-fixed state. The datasets were divided into training, validation, and test sets, with subjects distributed in an approximate ratio of 70:15:15, respectively (Table 2). To ensure the robustness of our results, we generated 5 different subject-wise data splits. For each of these splits, we conducted separate training and performance evaluation processes. This approach allows us to assess the model’s generalization ability across different subject groups and mitigate potential biases from any single data split.

### 4.2. Experimental Settings

In our experiments, we evaluate the performance of both MLP-based and CNN-based models. For MLP-based models, we employ MLP-Mixer and gMLP, examining their performance across a range of parameter configurations. As for CNN-based models, we adopt VGG16, ResNet-18, and EfficientNet-B0, maintaining their original architectures as proposed in their respective papers. This selection of models allows us to compare the performance of newer MLP-based approaches against well-established CNNs in the HAR field. For MLP-Mixer, we fix several hyperparameters based on the original paper; the patch resolution is set to P=16, and the number of Mixer layers is maintained at 12. For the three parameters that are the focus of our investigation, we use values based on those in the original paper:

- Hidden size: *C*;
- The output dimension of the token-mixing MLP layer: $D_{S}$;
- The output dimension of the channel-mixing MLP layer: $D_{C}$.

This approach allows us to establish a baseline configuration that closely aligns with the model’s original design for our subsequent experiments. Similar to our approach with MLP-Mixer, we apply the same methodology to gMLP. For gMLP, we fix several hyperparameters based on the original paper; the patch resolution is set to P=16, and the number of gMLP blocks is maintained at 30. For the two MLP parameters that are the focus of our investigation, we use values based on those in the original paper:

- The first MLP output dimensions in gMLP block and the number of channels for the input tokens: $d_{model}$;
- The second MLP output dimensions in gMLP block: $d_{ffn}$.

We investigate models with reduced MLP parameters at various percentages of the original configuration, considering the original count as 100%. We examine reduction rates of 75%, 50%, 25%, 12.5%, and 6.67% relative to the original number of parameters. Based on these reduction rates, we proportionally reduce the number of parameters that are the focus of our investigation. When applying CNN-based models to HAR, we used the same hyperparameters as specified in the original papers, while modifying the convolution operations to one-dimensional ones to accommodate sensor data. This modification resulted in a reduction in trainable parameters compared to the original papers. When applying MLP-based models to HAR, we maintained their structure as specified in the original papers to ensure a fair comparison with CNN-based models wherever possible. As the CNN-based models had reduced trainable parameters, we similarly evaluated MLP-based models by progressively reducing the number of units in MLP layers, which constitute their trainable parameters. Table 3 and Table 4 show the number of parameters and model sizes resulting from these reductions. Table 5 shows the number of model parameters for the CNN-based model. For deep learning models, the actual required memory capacity can be calculated by multiplying the number of parameters by the size of the data type. In this study, since we used half-precision floating-point numbers, the data type size is 4 [bytes]. Therefore, the memory consumption can be calculated by multiplying the number of parameters shown in the table by 4 [bytes].

The training parameters are as follows:

- Batch size: 256;
- Optimizer function: Adam;
- Learning rate: 0.0001;
- Epochs: 100.

The experiments were conducted on a computer equipped with a Intel Core i7-13700K processor, 64 GB RAM, and an NVIDIA GeForce RTX 4090.

## 5. Results

### 5.1. Accuracy and Model Parameters

**Figure 4:**
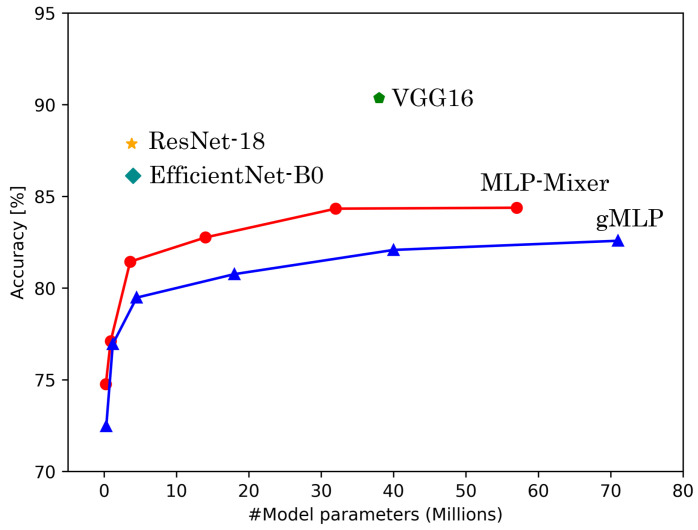
The number of model parameters vs. accuracy for HASC.

**Figure 5:**
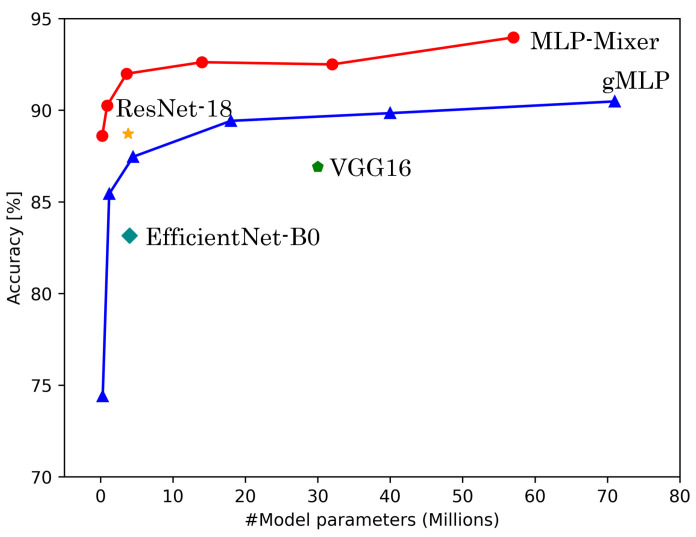
The number or model parameters vs. accuracy for UCI HAR.

**Figure 6:**
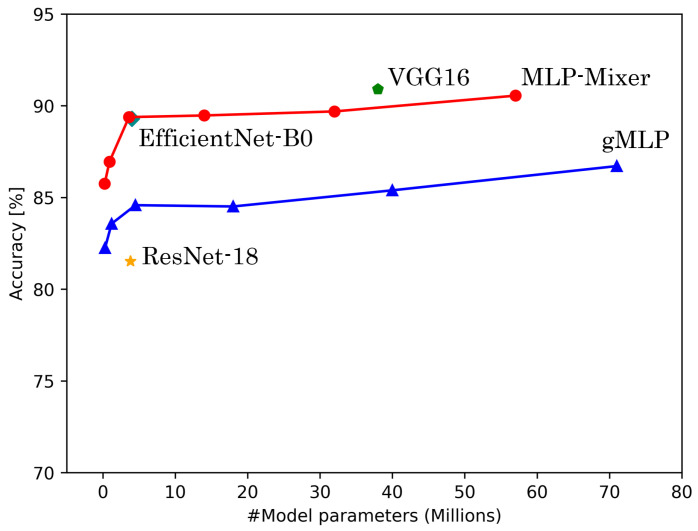
The number of model parameters vs. accuracy for WISDM.

**Figure 7:**
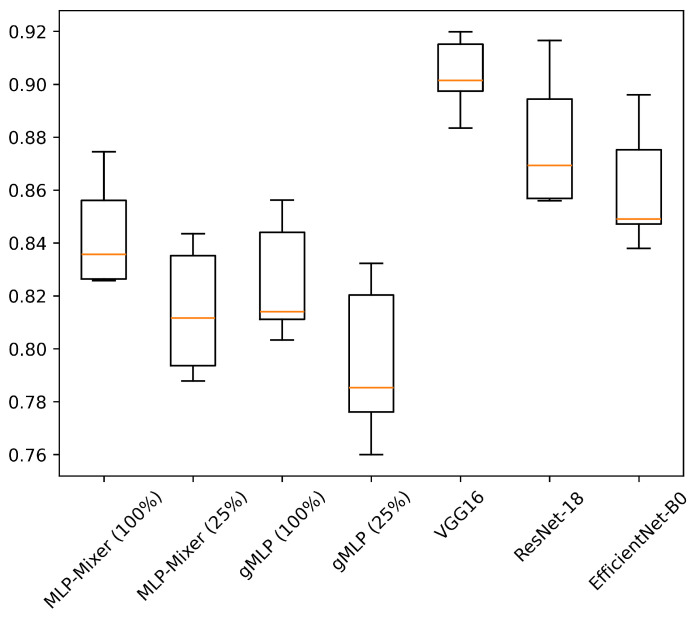
Accuracy variations across five experiments for each model on the HASC. The value in “( )” after each model name indicates the reduction rate.

**Figure 8:**
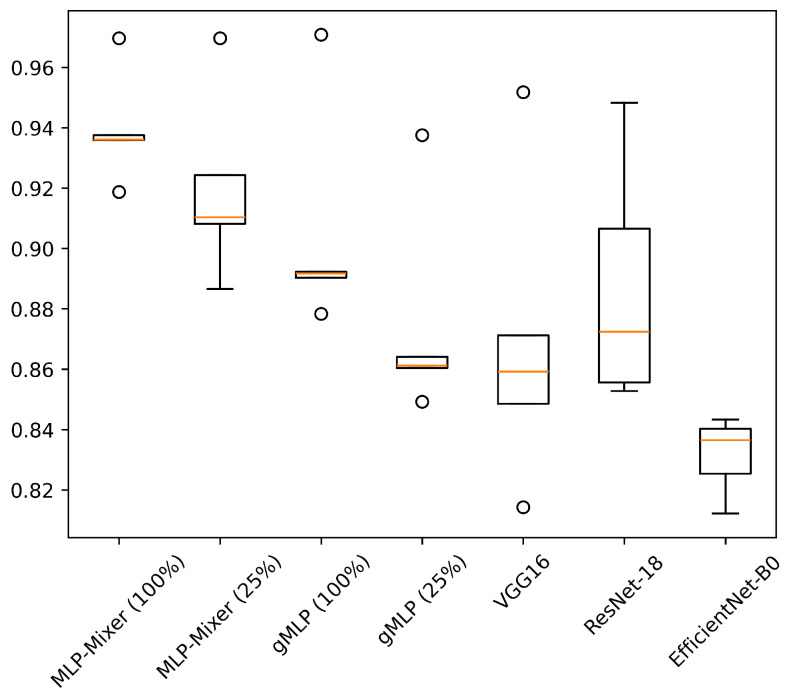
Accuracy variations across five experiments for each model on the UCI HAR. The value in “( )” after each model name indicates the reduction rate.

**Figure 9:**
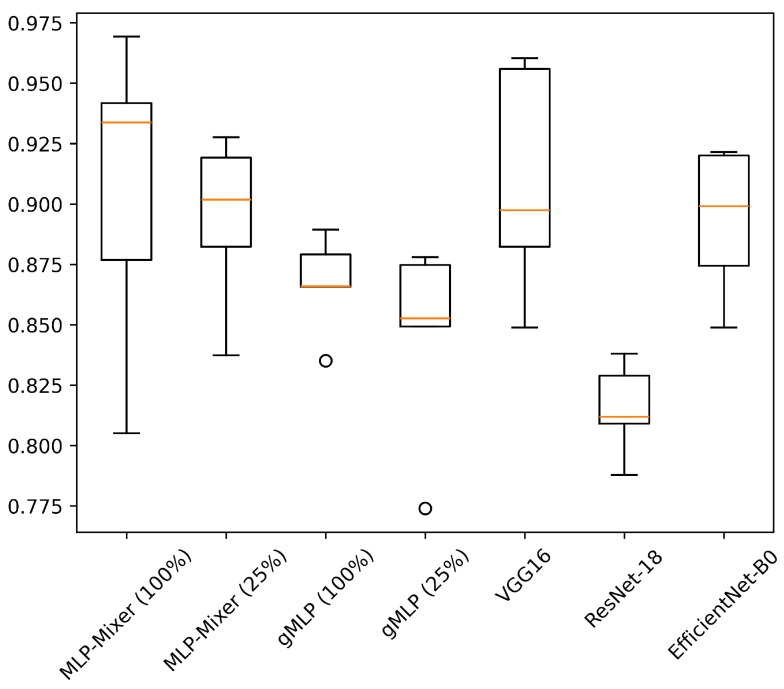
Accuracy variations across five experiments for each model on the WISDM. The value in “( )” after each model name indicates the reduction rate.

Table 6 shows the results of experiments conducted on HASC, UCI HAR, and WISDM datasets. Figure 4, Figure 5 and Figure 6 show the relationship between model parameters and accuracy. We report the accuracy and macro-F1 score as means ± standard deviations from experiments using 5 data patterns for each dataset. As we conducted five experiments for each model, Figure 7, Figure 8 and Figure 9 show the variations in accuracy. For MLP-Mixer and gMLP, we show the results for both the original parameters and the case where parameters are reduced (reduction rate = 25%) to match those of ResNet-18 and EfficientNet-B0.

**Figure 10:**
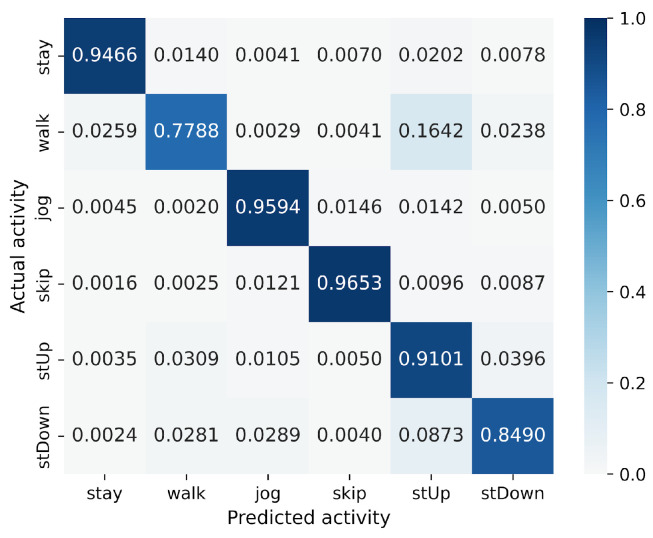
Confusion matrix of models for the HASC (VGG16).

**Figure 11:**
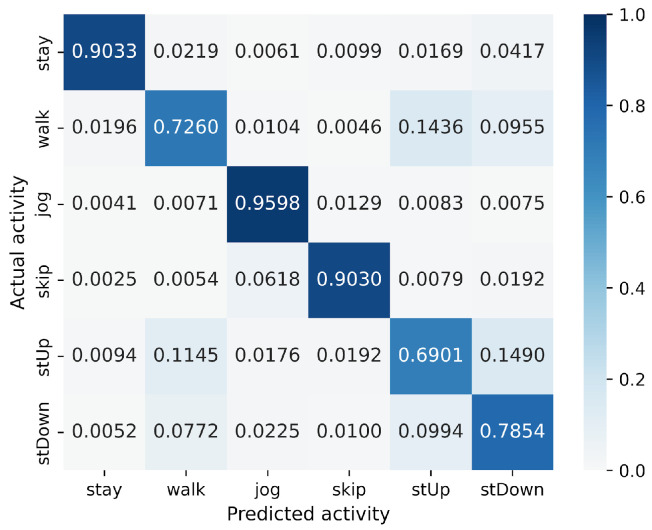
Confusion matrix of models for the HASC (MLP-Mixer (reduction rate = 100%)).

**Figure 12:**
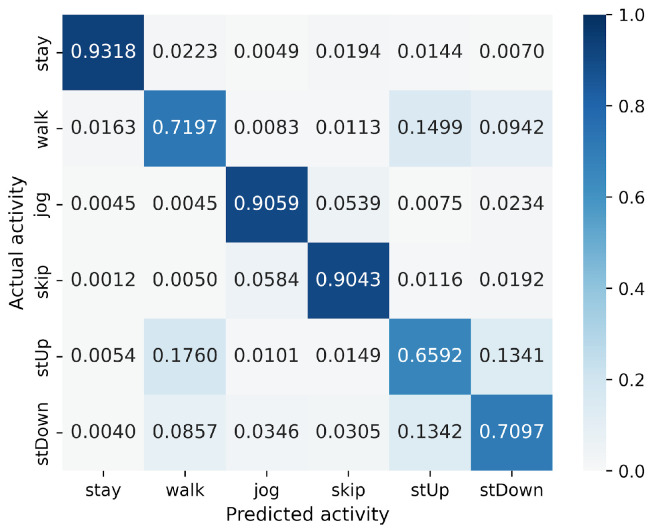
Confusion matrix of models for the HASC (gMLP (reduction rate = 100%)).

First, we examine the results for the HASC dataset. The MLP-based models perform worse than the CNN-based models. Among CNN-based models, VGG16 achieves the highest accuracy of 90.36%. In contrast, for MLP-based models, MLP-Mixer (base) shows the highest accuracy at 84.38%, which is 6% lower than the best CNN-based model. Figure 4 shows that CNN-based models also prove superior in analyzing the relationship between model parameters and accuracy. To analyze the inferior accuracy of MLP-based models compared to VGG16, we present confusion matrices in Figure 10, Figure 11 and Figure 12. These matrices summarize the predictions made by VGG16, MLP-Mixer, and gMLP (both at 100% parameter count) on the same data, alongside the actual correct activity. The matrices reveal that all models frequently misclassify “walk” activities, often confusing them with “stUp” or “stDown”. For the “stUp” activity, VGG16 demonstrates good recognition performance, while both MLP-based models exhibit higher error rates, often mistaking “stUp” for “walk” or “stDown”. Notably, gMLP performs particularly poorly in recognizing the “stDown” activities. These results indicate that MLP-based models struggle with distinguishing between “stUp” and “stDown” activities in the HASC dataset. This suggests that MLPs may have difficulty extracting the necessary features for these specific activities.

**Figure 13:**
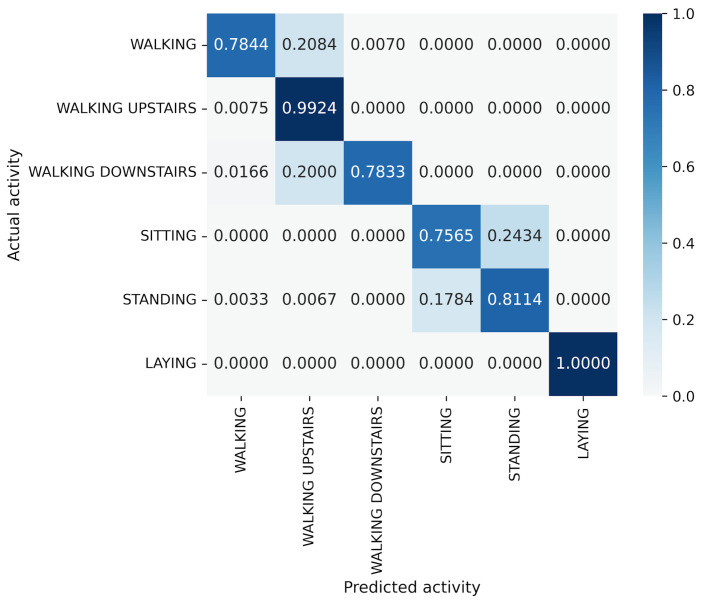
Confusion matrix of models for the UCI HAR (ResNet-18).

**Figure 14:**
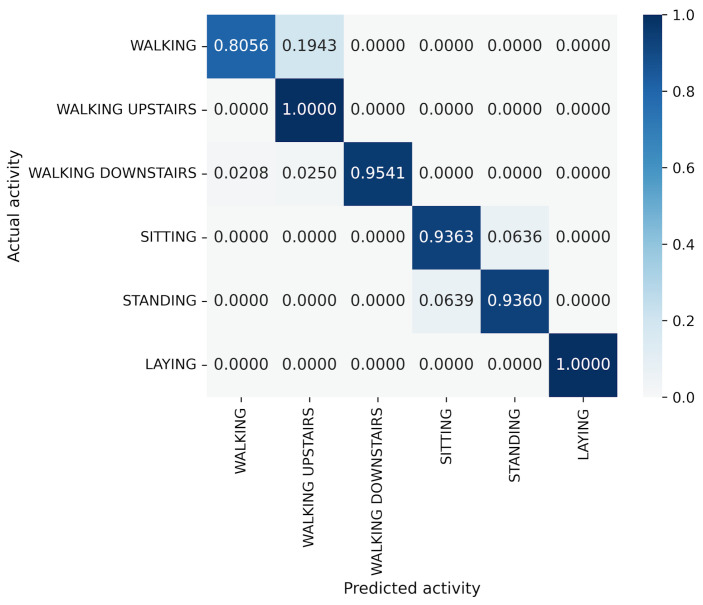
Confusion matrix of models for the UCI HAR (MLP-Mixer (reduction rate = 100%)).

**Figure 15:**
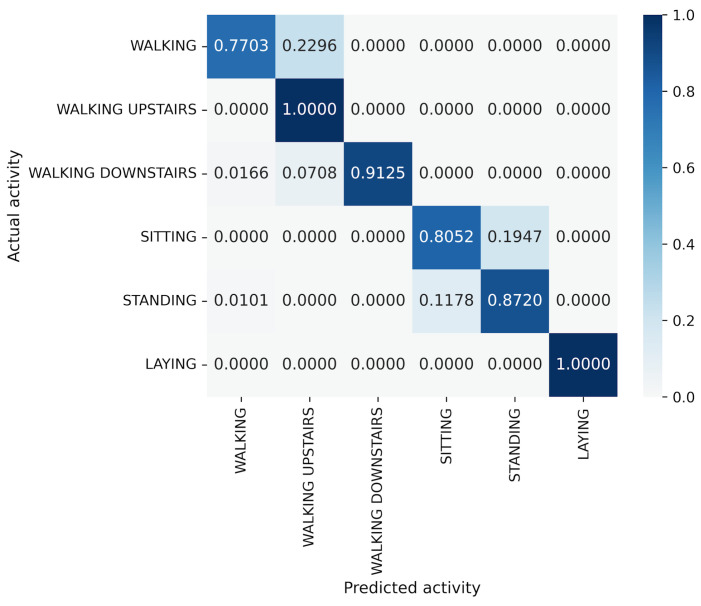
Confusion matrix of models for the UCI HAR (gMLP (reduction rate = 100%)).

For the UCI HAR dataset, MLP-Mixer outperforms other models in terms of both accuracy and parameter efficiency (Figure 5). Furthermore, gMLP surpasses both VGG16 and EfficientNet-B0. The best model, MLP-Mixer, achieves 93.97% accuracy, which is 5% higher than 88.72% of ResNet-18, the top performer among CNN-based models. To analyze the inferior accuracy of MLP-based models compared to ResNet-18, we present confusion matrices in Figure 13, Figure 14 and Figure 15. To analyze the results in detail, we examine the confusion matrices summarizing the activities predicted by ResNet-18, MLP-Mixer (reduction rate = 100%), and gMLP (reduction rate = 100%) for the same data, along with the actual correct activities. All models achieved high accuracy for “WALKING UPSTAIRS” and “LAYING” activities. Furthermore, for the “WALKING DOWNSTAIRS” activity, both MLP-based models, MLP-Mixer and gMLP, demonstrated superior recognition performance compared to ResNet-18. Focusing on the MLP-Mixer results, we observe superior performance in recognizing all activities compared to both other models, suggesting that MLP-Mixer can extract features essential for recognition from the UCI HAR dataset. Given the high accuracy at the baseline reduction rate of 100%, it is conceivable that even with a reduction in the number of parameters, MLP-Mixer may maintain its performance superiority over other models. Focusing on gMLP, we observe improved accuracy compared to ResNet-18 for all activities except “WALKING”. However, the margin of improvement is smaller when compared to that of MLP-Mixer. Consequently, we suggest that the gradual decrease in accuracy due to parameter reduction may have resulted in inferior performance per parameter compared to ResNet-18.

**Figure 16:**
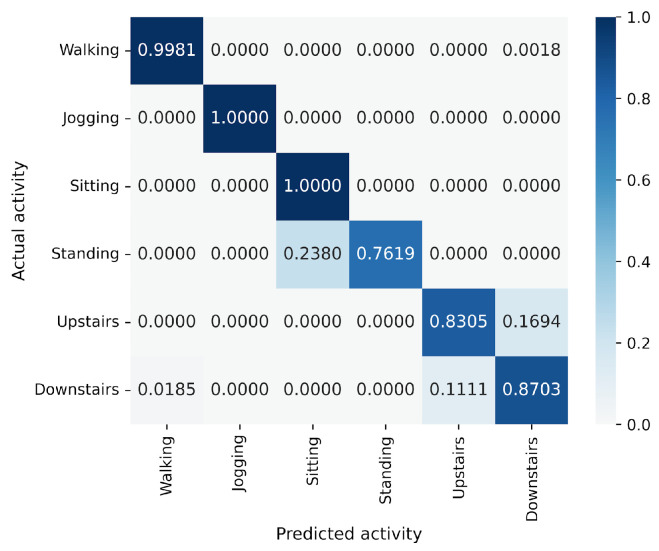
Confusion matrix of models for the WISDM (VGG16).

**Figure 17:**
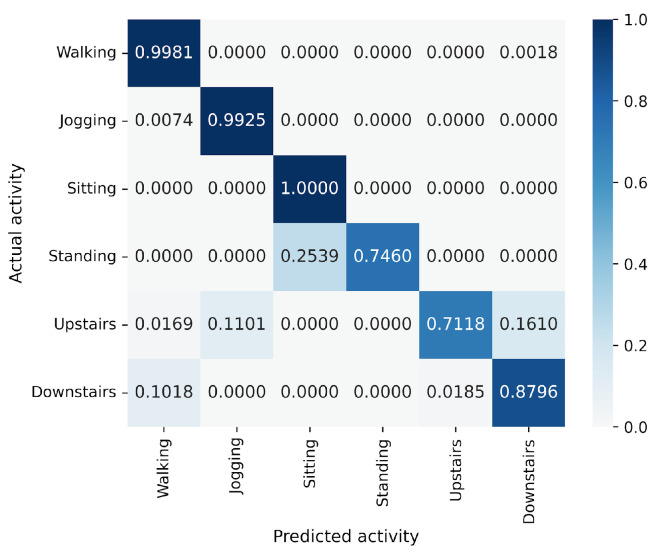
Confusion matrix of models for the WISDM (MLP-Mixer (reduction rate = 100%)).

**Figure 18:**
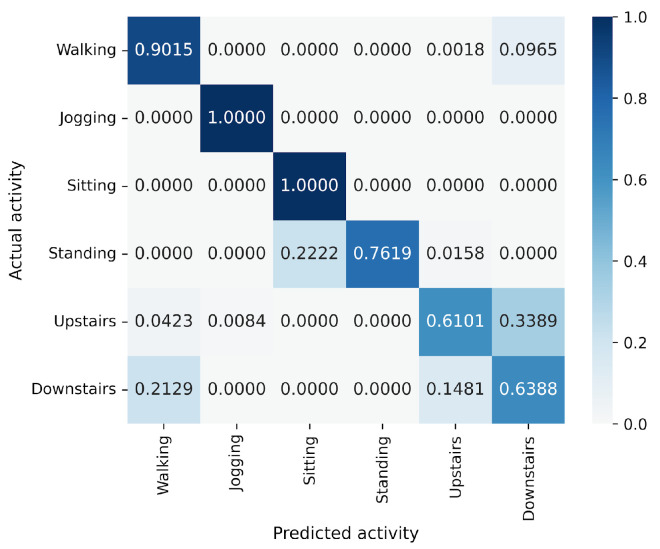
Confusion matrix of models for the WISDM (gMLP (reduction rate = 100%)).

In the WISDM results, VGG16 achieves the best accuracy at 90.91%, followed closely by MLP-Mixer at 90.55%, which is slightly lower than VGG16 (Figure 6). The number of parameters for MLP-Mixer and VGG16 are 30 M and 57 M, respectively. This indicates that the performance per parameter of MLP-Mixer is lower than that of VGG16, despite MLP-Mixer having fewer parameters. Conversely, MLP-Mixer demonstrates higher performance relative to the number of model parameters compared to ResNet-18 and EfficientNet-B0. gMLP performs better than ResNet-18 but falls short of VGG-16 and EfficientNet-B0. To analyze the inferior accuracy of MLP-based models compared to VGG16, we present confusion matrices in Figure 16, Figure 17 and Figure 18 of MLP-Mixer (reduction rate = 100%), and gMLP (reduction rate = 100%) for the same data, along with the actual correct activities. High accuracy is achieved for “Walking”, “Jogging”, and “Sitting” activities across all models. In contrast, the accuracy for “Standing”, “Upstairs”, and “Downstairs” activities is comparatively lower across the models. Focusing on MLP-Mixer, we observe that it achieves accuracy rates comparable to VGG16 for “Standing” and “Downstairs” activities. However, its performance on the “Upstairs” activity is notably lower. This underperformance in recognizing the “Upstairs” activity contributes to the slightly lower overall accuracy of MLP-Mixer compared to VGG16. Focusing on the gMLP, the accuracy rates for “Upstairs” and “Downstairs” activities are approximately 10% to 20% lower than those of other models. This underperformance in these specific activities contributes to the lower overall accuracy of gMLP compared to other models.

Overall, among MLP-based models, MLP-Mixer consistently outperforms gMLP. Conversely, gMLP tends to underperform compared to existing CNN-based models when applied to datasets other than UCI HAR. MLP-Mixer-based models show a clear trend where accuracy changes proportionally to the model parameters. This indicates that the number of model parameters can be flexibly changed and applied according to the purpose, such as using large model parameters when high accuracy is required and fewer model parameters when efficiency, such as inference speed, is desired. We obtain similar results for CNN-based models adapted from image classification using one-dimensional convolution, where outcomes vary for each dataset. These results are consistent with those obtained in an existing study [18]. Therefore, it is essential to construct appropriate models for each target data and problem, including MLP-based models. Performance differences across datasets are observed for all models. This can be attributed to variations in data collection methods, sampling rates, and window sizes set for experiments. To account for data imbalance, we examine the macro-F1 scores in Table 6. We can confirm that higher accuracy corresponds to higher macro-F1 scores. The macro-F1 scores are similar to accuracy in most cases, indicating no significant bias toward specific classes. On the other hand, for WISDM, all models show macro-F1 scores that are several points lower than their accuracy. This can be attributed to certain activities with poor recognition performance having a more pronounced impact than in other datasets, as evident from the confusion matrices. This can be attributed to a greater influence of specific activities with poor recognition performance than in other datasets.

### 5.2. Training Time and Latency

We measured the training time for each model using WISDM. The results, including theoretical computational complexity in FLOPs, are shown in Table 7. For MLP-Mixer and gMLP, we observed that training time increased proportionally with FLOPs. When examining all models, we found that training time was not necessarily proportional to FLOPs. For example, VGG16, which has the highest FLOPs, showed a shorter training time than other models. This can be attributed to computational optimizations through libraries such as cuDNN on the software side. On the hardware side, it is likely due to the acceleration of specific calculations by CUDA cores and Tensor Cores in the GPU. We also measured latency, which is the inference time per data point, and found that all models performed at approximately 2 ms. This lack of noticeable speed differences is likely attributable to the extremely high-performance GPU in our experimental environment.

### 5.3. Limitations

In this study, we applied MLP-Mixer and gMLP, models originally proposed for image recognition, to the HAR domain using three ADL datasets. We confirmed the effectiveness of MLP-Mixer and gMLP by demonstrating their comparable performance to representative CNN models that were originally proposed for image recognition and have been established as effective in the HAR. However, we have not determined the relative advantages and disadvantages compared to various HAR-specialized models presented in related works. In our experiments, to maintain fairness in comparison with CNN models, we kept the architectures and hyperparameters of MLP-Mixer and gMLP as close as possible to their original proposals. Therefore, we have not demonstrated that the hyperparameters of MLP-Mixer and gMLP adopted in this paper are necessarily optimal for HAR.

## 6. Conclusions

This study has applied MLP-based models, specifically MLP-Mixer and gMLP, to HAR and evaluated their performance. We have revealed the accuracy when gradually reducing the model parameters for both models and obtained results showing that accuracy is proportional to the model parameters. Our experiments demonstrated that MLP-based models, especially MLP-Mixer, sometimes outperform existing CNN-based models. The experimental results indicate that MLP-based models are not inferior to existing CNNs in performance and can be competitive. In the future, we aim to improve performance by adding various regularization techniques and explore the possibility of enhancing performance by combining CNN-based models with MLP-based models [41].

## Figures and Tables

**Table 1:** Datasets.

Dataset	Sampling Freq. [Hz]	# Subjects	# Activities	Activities	Data Dimension
HASC	100	185	6	stay, walk, jog, skip, stUp, stDown	(256, 3)
UCI HAR	50	30	6	walking, walking upstairs, walking downstairs, sitting, standing, laying	(128, 3)
WISDM	20	36	6	Walking, Jogging, Upstairs, Downstairs, Sitting, Standing	(256, 3)

**Table 2:** Number of subjects in the case of splitting the subjects into three categories.

Dataset	# Train Subjects	# Validation Subjects	# Test Subjects
HASC	129	28	28
UCI HAR	20	5	5
WISDM	26	5	5

**Table 3:** The number of parameters in MLP-Mixer.

Reduction Rate	Hidden Size: *C*	MLP dim.: $D_{S}$	MLP dim.: $D_{C}$	# Params
100%	768	384	3072	57 M
75.0%	576	288	2304	32 M
50.0%	384	192	1536	14 M
25.0%	192	96	768	3.6 M
12.5%	96	48	384	0.91 M
6.67%	48	24	192	0.24 M

**Table 4:** The number of parameters in gMLP.

Reduction Rate	MLP dim.: $d_{\mathrm{model}}$	MLP dim.: $d_{\mathrm{ffn}}$	# Params
100%	512	3072	71 M
75.0%	384	2304	40 M
50.0%	256	1536	18 M
25.0%	128	768	4.5 M
12.5%	64	384	1.2 M
6.67%	32	192	0.30 M

**Table 5:** The number of model parameters in CNN-based models.

Model	# Params
VGG16	38 M
ResNet-18	3.8 M
EfficientNet-B0	4.0 M

**Table 6:** Accuracy of each model for 3 datasets.

Model	Reduction Rate	Accuracy [%]			Macro-F1 Score [%]		
		HASC	UCI HAR	WISDM	HASC	UCI HAR	WISDM
VGG16	-	90.36 ± 1.30	86.91 ± 4.56	90.91 ± 4.31	90.72 ± 1.23	87.82 ± 4.62	87.72 ± 5.96
ResNet-18	-	87.87 ± 2.35	88.72 ± 3.61	81.52 ± 1.74	88.25 ± 2.47	89.57 ± 3.42	77.34 ± 1.80
EfficientNet-B0	-	86.12 ± 2.14	83.16 ± 1.14	89.29 ± 2.78	86.65 ± 1.93	84.14 ± 1.26	83.92 ± 4.32
MLP-Mixer	100%	84.38 ± 1.89	93.97 ± 1.65	90.55 ± 5.85	84.75 ± 1.97	94.32 ± 1.71	87.28 ± 8.67
	75.0%	84.33 ± 2.24	92.50 ± 2.57	89.69 ± 4.77	84.62 ± 2.33	92.89 ± 2.82	86.85 ± 6.06
	50.0%	82.76 ± 2.11	92.62 ± 3.10	89.47 ± 4.95	83.33 ± 2.23	93.12 ± 3.23	86.09 ± 5.32
	25.0%	81.44 ± 2.20	91.99 ± 2.77	89.38 ± 3.22	81.69 ± 2.44	92.44 ± 2.92	84.27 ± 4.11
	12.5%	77.11 ± 2.14	90.25 ± 3.46	86.95 ± 3.35	77.60 ± 2.32	90.82 ± 3.78	81.20 ± 3.98
	6.67%	74.76 ± 2.06	88.61 ± 3.13	85.75 ± 3.99	75.30 ± 2.20	89.19 ± 3.27	80.69 ± 4.21
gMLP	100%	82.58 ± 2.06	90.48 ± 3.35	86.71 ± 1.83	83.02 ± 2.17	91.33 ± 3.38	81.16 ± 2.90
	75.0%	82.08 ± 2.27	89.84 ± 4.04	85.39 ± 2.89	82.50 ± 2.59	90.54 ± 4.31	79.26 ± 3.87
	50.0%	80.76 ± 2.48	89.42 ± 3.91	84.51 ± 2.44	81.25 ± 2.62	90.32 ± 3.94	80.13 ± 4.01
	25.0%	79.48 ± 2.72	87.46 ± 3.19	84.58 ± 3.77	80.02 ± 2.88	88.50 ± 3.20	78.45 ± 3.58
	12.5%	76.95 ± 2.42	85.44 ± 3.89	83.57 ± 2.82	77.95 ± 2.57	86.25 ± 4.09	77.69 ± 4.25
	6.67%	72.47 ± 2.76	74.40 ± 3.23	82.25 ± 2.99	73.87 ± 3.00	67.34 ± 7.04	70.57 ± 4.26

**Table 7:** Training time and latency for WISDM.

Model	Reduction Rate	# FLOPs	Training Time [s]
VGG16	-	178 M	27
ResNet-18	-	44 M	21
EfficientNet-B0	-	49 M	264
MLP-Mixer	100%	57 M	353
	75%	32 M	186
	50%	14 M	99
	25%	4 M	49
	12.5%	1.0 M	42
	6.67%	0.3 M	30
gMLP	100%	71 M	375
	75%	40 M	231
	50%	18 M	140
	25%	5 M	81
	12.5%	1.2 M	75
	6.67%	0.3 M	74

## Data Availability

The original contributions presented in this study are included in the article. Further inquiries can be directed to the corresponding author.
